# Renal deficit and associated factors in children born with low birth weight

**DOI:** 10.1590/2175-8239-JBN-2022-0154en

**Published:** 2023-04-03

**Authors:** Marynéa Silva do Vale, Patrícia Franco Marques, Milady Cutrim Vieira Cavalcante, Mateus Noleto Brito, Alcione Miranda dos Santos, Natalino Salgado-Filho, José Luiz M. B. Duarte

**Affiliations:** 1 Universidade Federal do Maranhão, Hospital Universitário, São Luís, MA, Brazil.; 2 Universidade Federal do Maranhão, Departamento de Medicina I, São Luís, MA, Brazil.; 3 Universidade Federal do Maranhão, Departamento de Saúde Pública, São Luís, MA, Brazil.; 4 Universidade do Estado do Rio de Janeiro, Departamento de Pediatria, Rio de Janeiro, RJ, Brazil.

**Keywords:** Infant, Infant, Low Birth Weight, Glomerular Filtration Rate, Kidney Function Tests, Creatinine, Cystatin C, Lactente, Recém-Nascido de Baixo Peso, Taxa de Filtração Glomerular, Testes de Função Renal, Creatinina, Cistatina C

## Abstract

**Introduction:**

Kidney problems may be due to low birth weight alone or may occur in association with other conditions. The objective this study was to evaluate the association between maternal and birth characteristics, anthropometric measurements, and kidney function deficit in low birth weight infants.

**Methods:**

Cross-sectional study with children who were born weighing < 2500 grams and were under outpatient follow-up. Maternal factors investigated were prenatal care and presence of hypertension, diabetes, and infection during pregnancy. The children’s variables were sex, gestational age, birth weight, Apgar score, use of nephrotoxic medications, age, body weight at the time of evaluation, height, and serum creatinine and cystatin C dosages. The glomerular filtration rate (GFR) was estimated with the combined Zapittelli equation. Multivariate logistic regression model was used for identification of associated factors, with renal function deficit (GFR < 60 mL/min/1.73 m^2^) as the dependent variable.

**Results:**

Of the 154 children evaluated, 34.42% had kidney function deficit. Most of them had a gestational age > 32 weeks (56.6%), a mean birth weight of 1439.7 grams, and mean estimated GFR of 46.9 ± 9.3 mL/min/1.73 m^2^. There was a significant association of GFR < 60 mL/min/1.73 m^2^ with children’s current weight and use of nephrotoxic drugs.

**Discussion:**

Children born with low birth weight had a high prevalence of kidney function deficit and current normal weight was a protective factor while the use of nephrotoxic drugs during perinatal period increased the chance of kidney deficit. These findings reinforce the need to evaluate the kidney function in these children, especially those who use nephrotoxic drugs.

## RESUMO

**Introdução:**

Problemas renais podem ser devido apenas ao baixo peso ao nascer ou podem ocorrer em associação com outras condições. O objetivo deste estudo foi avaliar a associação entre características maternas e de nascimento, medidas antropométricas e déficit da função renal em bebês de baixo peso ao nascer.

**Métodos:**

Estudo transversal com crianças que nasceram com peso < 2500 gramas e estavam sob acompanhamento ambulatorial. Os fatores maternos investigados foram cuidados pré-natal e presença de hipertensão, diabetes e infecção durante a gravidez. As variáveis das crianças foram sexo, idade gestacional, peso ao nascer, índice Apgar, uso de medicamentos nefrotóxicos, idade, peso corporal no momento da avaliação, altura e dosagens séricas de creatinina e cistatina C. A taxa de filtração glomerular (TFG) foi estimada com a equação combinada de Zapittelli. Utilizou-se um modelo de regressão logística multivariada para identificação de fatores associados, com déficit da função renal (TFG < 60 mL/min/1,73 m^2^) como variável dependente.

**Resultados:**

Das 154 crianças avaliadas, 34,42% apresentaram déficit da função renal. A maioria tinha idade gestacional > 32 semanas (56,6%), peso médio ao nascer de 1439,7 gramas, e TFG média estimada de 46,9 ± 9,3 mL/min/1,73 m^2^. Houve uma associação significativa da TFG < 60 mL/min/1,73 m^2^ com o peso atual das crianças e o uso de medicamentos nefrotóxicos.

**Discussão:**

Crianças nascidas com baixo peso apresentaram alta prevalência de déficit da função renal e o peso atual normal foi um fator de proteção, enquanto o uso de medicamentos nefrotóxicos durante o período perinatal aumentou a chance de déficit renal. Estes achados reforçam a necessidade de avaliar a função renal destas crianças, especialmente aquelas que usam medicamentos nefrotóxicos.

## Introduction

Low birth weight (LBW) is considered a major public health problem with prevalence around 15% to 20% among all births worldwide^1^. There is considerable variation in the prevalence of LBW across regions and within countries^1^, but most LBW births occur in low- and middle-income countries, especially in the most economically vulnerable populations^2^. In Brazil, the annual prevalence of LBW was about 8.5%^3^ between 2014 and 2018.

LBW is a predictor of adverse health outcomes, with short- and long-term consequences such as chronic disease in adulthood. This was the basis of the DOHaD studies - Origins of Health and Illness Development, which originated the hypothesis of Barker et al.^4^.

According to Barker, pregnancy and early life are periods when events occur that can influence genetic programming and cause disease in adulthood^4^. In this line of research, Luyckx et al. studied human nephrogenesis and the low endowment of nephrons in preterm infants. Nephrogenesis is completed around 34–37 weeks of gestation, after which nephrons no longer form. Therefore, preterm infants have immature kidneys and develop compensatory mechanisms with glomerular hyperfiltration, causing wear and loss of function^5^.

Other studies support the hypothesis of early life-related kidney disease in adulthood, as kidneys can be structurally and functionally altered by adverse events, as is the case with individuals born with LBW and have fewer nephrons^5,6^. Al Salmi and Hannawi^7^ point out that reduced nephron endowment arising from LBW occurs especially in more deprived populations.

The main causes of kidney function changes among children with LBW may be related to prenatal, perinatal, and postnatal factors^8^. Intrauterine changes can have repercussions on smaller kidneys, and the correlation between birth weight and number of nephrons or glomerular mass has been pointed out in different studies^8,9^. This can have repercussions on the development of kidney disease and more rapid deterioration of kidney function in patients with underlying kidney disease^8^. Other structural changes caused by adverse fetal and neonatal environment include peritubular and glomerular capillary thinning and low podocyte endowment, exacerbated by focal glomerulosclerosis and postnatal tubulointerstitial fibrosis^6^.

The factors that determine LBW are many and nonspecific, and may be associated with either premature birth^10^ or being small for gestational age (SGA), or both, and all are associated with increased risk of kidney disease^7,8,11,12,13^.

Environmental, hereditary, and maternal health factors during pregnancy may also influence the number of nephrons in individuals^4,5,14^. Low number of nephrons is related to factors that affect disadvantaged populations the most such as pre-eclampsia, diabetes in pregnancy, maternal overweight/obesity, maternal malnutrition^5,14^, advanced maternal age, teenage pregnancy^15,16^, consumption of nonsteroidal anti-inflammatory drugs (NSAIDs) during pregnancy^17^, and maternal drug exposure^18^.

In addition, kidney injuries in the neonatal phase may also be considered predisposing factors and include respiratory distress syndrome, low Apgar score^17^, neonatal acute kidney injury (AKI), nephrotoxic drugs, kidney hypoxia, and urinary tract infection^6^.

There is a gap in knowledge regarding the role of maternal characteristics and conditions involved in LBW in the development of kidney disease^5,14–16,18,19^ which, in addition to the complexity of this relationship, makes it difficult to identify newborns to monitored for kidney disease evaluation throughout life.

Thus, the objective of this study was to evaluate the association between maternal and birth characteristics, anthropometric measurements, and kidney function deficit in children born with low birth weight and identify increased risk of developing kidney disease.

## Methods

A cross-sectional study was conducted with children aged 6 months to 6 years born with low birth weight, who were followed at the neonatal intensive care unit (NICU) outpatient clinic of a tertiary public hospital located in northeast Brazil.

The population were children born from January 2014 to May 2018 weighing less than 2500 grams, who were admitted to and followed-up in the NICU outpatient clinic.

Children who returned at least once to the follow-up clinic from June 2017 to December 2021 were randomly selected. Twin children and children with congenital malformations of the kidney or urinary tract identified by intrauterine diagnosis or after birth in the Neonatal Unit were not included.

**Figure 1.**
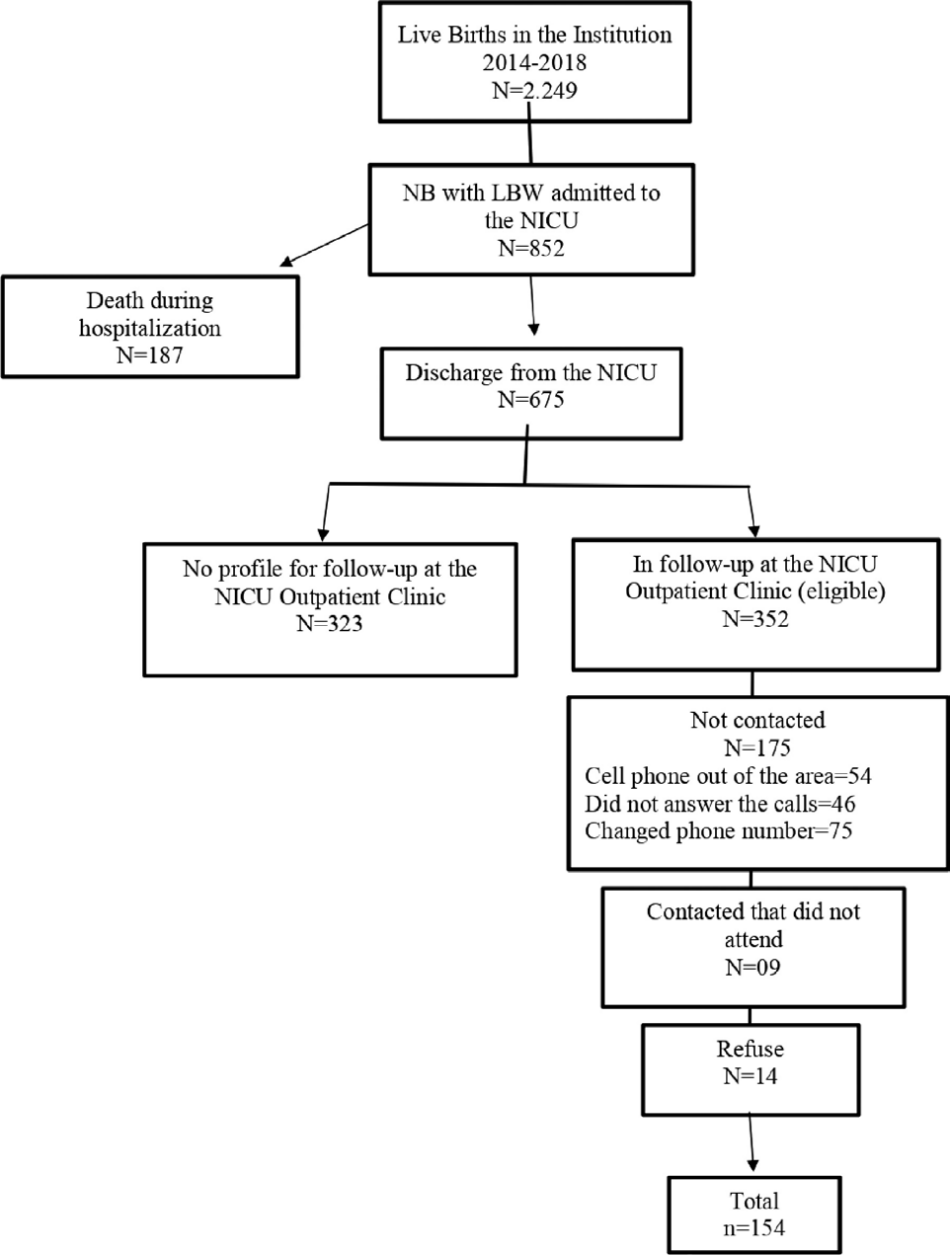
Flowchart.

From January 2014 to December 2018, 2,294 LBW children were born in the institution; 853 were admitted to the NICU and 178 died during hospitalization; 675 were discharged, and 352 were referred to the outpatient clinic. Of these, it was not possible to contact 175 families; 14 families did not accept to participate in the study and 9 were contacted but did not show up on the scheduled day. Contact difficulties were exacerbated due to the COVID-19 pandemic. A total of 154 children remained in the study (Figure 1).

The variables of interest were maternal characteristics during pregnancy, clinical information of the child at birth and during hospitalization, and anthropometric and clinical-laboratorial assessments obtained during routine consultations of the child in the outpatient clinic. Maternal and newborn-related data at birth were obtained from electronic medical records, which were recorded from June 2017 to December 2021 on a structured questionnaire prepared by the researchers.

The maternal variables were prenatal care (yes/no), number of prenatal visits, presence of diabetes mellitus (yes/no) and/or hypertension (yes/no), and pregnancy infections including syphilis, toxoplasmosis, rubella, cytomegalovirus, or herpes.

The variables considered at birth were sex, gestational age (in weeks), birth weight (in grams), fifth minute Apgar score (ranging from 0 to 10, where values below 7 may reflect perinatal asphyxia), and use of nephrotoxic medications in the perinatal period (yes/no).

Gestational age was determined by best obstetric estimate, first trimester ultrasound examination followed by the date of last menstrual period (LMP) and physical examination of the newborn using the New Ballard method and categorized as less than or equal to 32 weeks and greater than 32 weeks.

Regarding the variables of the children in the NICU outpatient clinic, the following were collected: age (in years), body weight (in kilograms), height (in centimeters), serum cystatin C (mg/L), and serum creatinine (mg/dL).

Height was obtained using a Sanny^®^ acrylic stadiometer with an accuracy of 0.1 cm and maximum extension of two meters, divided into tenths of a centimeter. The child was in the supine position and the lower limbs were extended, with the head positioned in the fixed part of the stadiometer and the feet in the movable part. Body weight was measured with a GLICOMED^®^ scale.

For cystatin C and serum creatinine dosages, venous or arterial blood samples were collected by clinical laboratory technicians and processed in the Clinical Analyses Laboratory. Plasma creatinine dosage was determined by the modified Jaffé colorimetric method, and the spectrophotometric reading was measured at 512 nm wavelength. Serum cystatin C dosage was obtained using immunoturbidimetric assay with reaction intensification by latex particles, internationally standardized^20^. The aggregate was determined turbidimetrically at 700/546 nm. Cystatin C was measured using Roche/Hitachi cobas C systems, with cobas C 311/501 automated analyzers (PETIA) using the Roche/Hitachi CYSC2 reagent. The frequency of calibrations occurred after reagent lot change or every 90 days.

The assays had international standardization, enabling IDMS (isotope dilution mass spectrometry) tracking^21^. For collection and preparation of blood samples, tubes with serum separation gel were used, centrifuged, and stored at –10 °C, and the assay was performed according to the manufacturer’s protocol.

Glomerular filtration rate (GFR) was measured in mL/min/1.73 m^2^ and estimated from the combined equation of Zapitelli et al.^22^:

$$\mathrm{GFR}=\frac{43.82\times e^{0.003\times \text{height(m)}}}{{\mathrm{CisC}}^{0.635}\times {\mathrm{CrP}}^{0.547}}$$

where CrP is plasma creatinine, expressed in mg/dL and CisC is cystatin C, expressed in mg/L. This formula was validated by Zappitelli et al.^22^ and showed good accuracy and sensitivity for predicting GFR in the pediatric population. In addition, scientific evidence points to better performance in the assessment of GFR when using cystatin and combined equations^23^.

Impaired kidney function was defined by GFR values below 60 mL/min/1.73 m^2^, as per KDIGO Guidelines. The GFR without any other evidence of kidney damage was chosen to classify deficit because values below this level represent a loss of at least 50% of normal kidney function^24^.

In the descriptive analysis, frequencies and percentages were calculated for categorical variables and median, first and third quartiles for numerical variables. The normality of the variables was assessed by the Shapiro-Wilk test. The chi-square test was used to compare categorical variables under study, and the Mann-Whitney test was used to compare numerical variables. In all tests, a 5% significance level was considered.

To identify the main factors associated with kidney function deficit, a logistic regression model was used. Odds ratios (OR) and 95% confidence intervals (95%CI) were also estimated. Statistical analyses were performed using Stata software, version 14.0.

This study was approved by the Research Ethics Committee under the consubstantiated opinion number 2.083.442 (CAAE 68490717.6.0000.5086).

## Results

The study included 154 children, of which 53 (34.4%) had kidney function deficit. Among these, most were male (56.6%), with gestational age greater than 32 weeks (56.6%), who did not need resuscitation, with a median Apgar score of 8, and a mean birth weight of 1,439.7 ± 347.5 grams. At the time of the evaluation, most of these children were aged between 12 and 24 months (43.4%), serum creatinine and cystatin levels were 0.5 ± 0.4 and 1.8 ± 0.4, respectively, and mean estimated GFR was 46.9 ± 9.3 mL/min/1.73 m^2^ (Table 1). The characteristics of the children who did not have altered kidney function are also described in Table 1.

**Table 1.** Characteristics of children born with low birth weight in a university hospital in northeastern brazil, from 2014 to 2018 according to presence or absence of altered kidney function

Variable	Altered kidney function (n = 154)						p-value
	No (n = 101)			Yes (n = 53)			
	n (%)	Md (Q1;Q3)	Average ± SD	n (%)	Md (Q1;Q3)	Average ± SD	
Sex							0.284
Female	53 (52.5)			23 (43.4)			
Male	48 (47.5)			30 (56.6)			
Gestational age (weeks)							0.067
≤ 32	29 (28.7)			23 (43.4)			
> 32	72 (71.3)			30 (56.6)			
Apgar 5th minute		8 (8;9)			8 (7;9)		0.877
Birth weight (grams)		1,230 (976;1.408)	1,243 ± 318		1,415 (1,266;1,660)	1,439 ± 347	< 0.001
Age of returns (months)							0.006
Adequacy weight x gestational age							
SGA	29 (65.9)			15 (34.1)			0.517
AGA	72 (66,1)			37 (33.9)			
LGA	0 (0,0)			1 (100,0)			
6 a 12	15 (14.9)			13 (24.5)			
13 a 24	26 (25.7)			23 (43.4)			
Above 24	60 (59.4)			17 (32.1)			
Return weight (kg)*		12.2 (9.9;15.0)	12.9 ± 3.9		10.3 (8.6;11.2)	10.5 ± 2.4	0.748
Height (centimeters)		90.0 (79.0;99.0)	89.4 ± 14.1		82.7 (75.3;86.0)	81.0 ± 10.7	0.001
Serum cystatin (mg/L)		1.1 (0.9;1.6)	1.2 ± 0.4		1.8 (1.6;2.0)	1.8 ± 0.4	0.001
Serum creatinine (mg/dL)		0.3 (0.2;0.4)	0.3 ± 0.1		0.5 (0.3;0.5)	0.5 ± 0.4	0.001
GFR (mL/min/1.73 m^2^)		77.4 (68.0;86.6)	79.8 ± 15.6		47.2 (41.2;55.7)	46.9 ± 9.3	0.001

*n less than 154. Md – median. Q1; Q3 – interquartile range. GFR – glomerular filtration rate. SGA – small for gestational age. AGA – appropriate for gestational age. LGA – large for gestational age.

Regarding maternal characteristics, the percentages of prenatal consultations among mothers of children with and without kidney deficit were 88.7% and 98.0%, respectively. Among the mothers of children with kidney deficit, 31.4% had infection during pregnancy, 11.8% had diabetes mellitus, and 27.5% had systemic arterial hypertension (Table 2).

**Table 2.** Characteristics of mothers of children born with low birth weight in a university hospital in northeastern brazil from 2014 to 2018 according to presence or absence of altered renal function

Variable	Alteration in kidney function		p-value
	No (n = 101)	Yes (n = 53)	
	n (%)	n (%)	
**Prenatal visits**			0.013
No	2 (2.0)	6 (11.3)	
Yes	99 (98.0)	47 (88.7)	
Pregnancy infection*			0.364
No	58 (61.1)	35 (68.6)	
Yes	37 (38.9)	16 (31.4)	
**Systemic arterial hypertension**			0.189
No	58 (61.7)	37 (72.5)	
Yes	36 (38.3)	14 (27.5)	
**Diabetes mellitus**			0.162
No	89 (94.7)	45 (88.2)	
Yes	5 (5.3)	6 (11.8)	

*n less than 154.

The association of maternal and child characteristics with renal function deficit is described in Table 3. There was a significant positive association between renal function deficit and current child weight (OR = 0.80; CI = 0.65–0.99). The use of nephrotoxic drugs during hospitalization was negatively associated with renal function (OR = 2.78; CI = 1.09–7.06) (Table 3).

**Table 3.** Association of altered kidney function with maternal characteristics and caracteristics of children born with low birth weight in a university hospital in northeastern brazil from 2014 to 2018

Variables	OR	p-value	CI
Male gender	0.75	0.53	0.30–1.84
Birth weight (grams)	1.00	0.12	0.99–1.00
Gestational age > 32 weeks	0.82	0.71	0.29–2.32
Apgar 5th minute	0.97	0.90	0.66–1.44
Use of nephrotoxic drugs	2.78	0.03	1.09–7.06
Age 12 to 24 months	1.58	0.49	0.42–5.88
Age above 24 months	1.84	0.42	0.42–8.09
Current weight (kg)	0.80	0.04	0.65–0.99
Presence of infection during pregnancy	0.65	0.36	0.25–1.64
Presence of systemic arterial hypertension	0.74	0.54	0.28–1.95
Presence of diabetes mellitus	1.40	0.75	0.17–11.46

OR – Odds Ratio. CI – Confidence Interval.

## Discussion

The children evaluated in this study showed high prevalence of kidney function deficit (34.4%) compared to international rates^7,11,13,25^. Children with higher weight at assessment had a lower chance of kidney function deficit (OR = 0.80; CI = 0.65–0.99), while the use of nephrotoxic drugs during hospitalization in Neonatal Unit increased the chance of this deficit (OR = 2.78; CI = 1.09–7.06), both with statistical significance.

Epidemiological information on kidney deficit in children of this age group who had LBW is scarce. A study developed in Japan to estimate the prevalence of pediatric CKD recruited individuals aged 3 months to 15 years born between 1993 and 2010 and identified a prevalence of 27.8% of CKD^13^. Other studies have indicated prevalence of kidney deficit among individuals born with LBW of 8% in Australia, 16.1% in Norway, and 23.2% in the US^7,11,25^. These are studies that performed long-term follow-up and identified kidney deficit in later ages.

The high prevalence found in our study (34.4%) may be related to the lower number of nephrons arising from causes that mainly affect disadvantaged populations^5,14–18^.

In this study, birth weight was not significantly associated and children with higher weight at assessment had a lower chance of kidney function deficit. The protective role of balanced catch-up nutrition for the development of chronic non-communicable diseases, including CKD, has been suggested in other studies, including the population of children aged 5–10 years who were born prematurely and with very low birth weight^9,19,26^, an age group similar to the present study.

Iyengar et al.^9^ conducted a cohort study with children evaluated at 6, 18, and 24 months with the goal of identifying the growth and kidney function of 100 newborns with LBW compared to 66 with normal weight. Among newborns with LBW, although kidney volume was significantly smaller at all three time points (p < 0.001), GFR was equivalent at 18 and 24 months, suggesting relative hyperfiltration in the smaller kidneys, which may be a precursor to the disease in adults.

Another prospective cohort study conducted in the Netherlands evaluated the impact of infant feeding on kidney function in 5,043 children with a mean age of 6 years and found that those who were never breastfed had smaller kidney volumes and lower estimated GFR (–2.42 mL/min/1.73 m^2^; 95% CI:–4.56; –0.28) while those who were breastfed had the shorter duration of breastfeeding associated with smaller kidney volume and lower risk of microalbuminuria (p < 0.05). These results suggest that breastfeeding is associated with subclinical changes in kidney outcomes in childhood^26^.

The protective effect of weight in this study may be related to the hospital feeding, such as early initiation of enteral diet, parenteral nutrition on the first day of life, and exclusive breastfeeding at hospital discharge, having positive repercussions on weight. Holzer et al.^19^ report that the pattern of nutritional recovery in the first year of life may be related to the protective effect of weight. It is known that human milk have a lower quantity but better quality of protein compared to infant formula, as well as lower concentrations of electrolytes, better bioavailability of micronutrients, and other factors such as long-chain polyunsaturated fatty acids, which may help reduce inflammation and protect kidney development^27^.

Another important result of this study was that children born with LBW who were exposed to the use of nephrotoxic drugs during their NICU hospitalization had a 2.781 times greater chance of kidney function deficit. This finding is supported by the literature^28^ in a study that found that the average number of medication courses for LBW children is higher, with the most used drugs being ampicillin, gentamicin, caffeine citrate, vancomycin, furosemide, fentanyl, dopamine, and midazolam^29^. The main nephrotoxic drugs used by the children in this study were gentamicin, furosemide, amikacin and ibuprofen.

It is of paramount importance that healthcare professionals be more rigorous about preventing nephrotoxicity by managing antimicrobial therapy and other nephrotoxic drugs according to GFR estimates.

The literature points to a reduction in the endowment of nephrons in LBW newborns associated with several perinatal factors such as health conditions during pregnancy, so that genetic factors, maternal nutritional status, diabetes mellitus, and preeclampsia among others can influence kidney development^4,5,14–16^. In this study, mother characteristics were not related to kidney deficit, but high prevalence of infection during pregnancy, diabetes mellitus and systemic arterial hypertension were related.

Although the findings of this study do not allow inferences to be drawn, they endorse the knowledge about the influence of extrauterine conditions to which LBW children are exposed and which may increase or reduce the chances of kidney function deficit. This is an important result related to better clinical practices, which include optimization of enteral nutrition such as use of breast milk during hospitalization and at hospital discharge and interventions aimed at better managing the administration of nephrotoxic drugs during intensive care.

Another strength of this study was the use of a combined equation of two biomarkers to estimate GFR, which performs better than equations based on one marker alone^30^ and is considered the most sensitive and specific parameter for detecting changes in kidney function in a pediatric population^23^.

A limitation of the study is that the children evaluated were followed-up in an outpatient clinic of a university hospital, thus the estimates are specific for this group and cannot be extrapolated to the entire population of that place. However, the findings of the study show the importance of monitoring the kidney function in LBW newborns, since studies in these children that evaluate GFR throughout the first years of life are still scarce. The absence of data related to dosage and timing of use of nephrotoxic medications may have been a limitation of this study, but this information is commonly collected in clinical studies focused on AKI^29^, which was not the subject of this study.

The findings of this study indicated a high prevalence of kidney deficit in children, revealing the need to monitor kidney function and evaluate the nutritional status of these children, especially those who used nephrotoxic drugs during the perinatal period.

These results reinforce the idea that postnatal factors may increase the vulnerability to kidney disease in LBW children and can be used to establish outpatient follow-up care protocols focused on early identification and timely referrals aimed at preventing kidney disease and its complications.

Further studies evaluating GFR throughout the early years of life in LBW children are needed to identify other risk factors for kidney disease, especially in populations living in socioeconomically disadvantaged conditions.
